# Mobility and Upright Posture Are Associated with Different Aspects of Cognition in Older Adults

**DOI:** 10.3389/fnagi.2016.00257

**Published:** 2016-11-08

**Authors:** Rajal G. Cohen, Anita N. Vasavada, Michelle M. Wiest, Maureen Schmitter-Edgecombe

**Affiliations:** ^1^Department of Psychology and Communication Studies, University of IdahoMoscow, ID, USA; ^2^Voiland School of Chemical Engineering and Bioengineering, Washington State UniversityPullman, WA, USA; ^3^Department of Integrative Physiology and Neuroscience, Washington State UniversityPullman, WA, USA; ^4^Department of Statistical Science, University of IdahoMoscow, ID, USA; ^5^Department of Psychology, Washington State UniversityPullman, WA, USA

**Keywords:** visuomotor, verbal memory, episodic memory, postural alignment, timed up and go, forward head posture, neck posture, gait

## Abstract

**Objectives**: Aging is associated with cognitive decline, including visuomotor and memory concerns, and with motor system changes, including gait slowing and stooped posture. We investigated the associations of visuomotor performance and episodic memory with motor system characteristics in healthy older adults.

**Methods**: Neurologically healthy older adults (*N* = 160, aged 50–89) completed a battery of cognitive and motor tasks. Cognitive variables were grouped by principal components analysis (PCA) into two components: visuomotor performance and verbal episodic memory. Our primary predictor variables were two aspects of motor function: timed-up-and-go (TUG) speed and neck angle. Additional predictor variables included demographic factors (age, sex and education) and indicators of physical fitness (body mass index/BMI and grip strength). All seven predictor variables were entered stepwise into a multiple regression model for each cognitive component.

**Results**: Poor visuomotor performance was best predicted by a combination of advanced age, high BMI and slow TUG, whereas poor verbal memory performance was best predicted by a combination of advanced age, male sex, low education and acute neck angle.

**Conclusions**: Upright posture and mobility were associated with different cognitive processes, suggesting different underlying neural mechanisms. These results provide the first evidence for a link between postural alignment and cognitive functioning in healthy older adults. Possible causal relationships are discussed.

## Introduction

Understanding the causes and correlates of age-related cognitive decline is important for developing methods to prevent and reverse that decline. One promising avenue of inquiry investigates relationships between motor functions and cognition. Most studies in this vein focus on a single aspect of motor function (such as mobility) and one or more aspects of cognition (such as attention; McGough et al., 2011; Donoghue et al., 2012). Here, we focused on two distinct cognitive domains in which age-related declines can impact everyday functioning: visuomotor performance and episodic memory. We investigated the associations of each cognitive domain with two aspects of motor function: mobility and upright posture.

Visuomotor performance involves the ability to synchronize visual information with physical movement of the body (Shumway-Cook and Woollacott, 2001). This constellation of skills is crucial for many everyday functions, such as navigating in a busy environment. Relevant laboratory measures are those that require integration between visual perception and motor skills for task completion, including spatial span and digit-symbol substitution as well as drawing, copying and constructive tasks. Visuomotor abilities are often weaker in older adults, especially those with cognitive decline. For instance, in a study using the digit-symbol substitution task young subjects (aged 18–40) completed 50% more items than older subjects (aged 60–88) within a 90 s time frame (Henry and Phillips, 2006). Performance on both backward and forward spatial span tasks shows marked decline with age, and spatial span declines more sharply than digit span (Myerson et al., 2003). In addition, poor performance on a design fluency task has been associated with pathological cognitive aging, when broadly defined (Chi et al., 2012).

Episodic memory is a subcategory of declarative memory. It describes memories that can be explicitly stated and that are tied to a particular time and place. Episodic memory is important for the accurate completion of many everyday tasks that support independent living (e.g., managing a schedule and medications). Episodic memory ability is most commonly measured in the laboratory using list and story learning and memory tasks. Declines in episodic memory with aging are well-documented and have been associated with conversion to dementia, (e.g., Silva et al., 2013). Data from two large-scale longitudinal studies that used a variety of episodic memory indicators showed that 3-year changes in episodic memory were modest in a sample of normal older adults (Dixon et al., 2004). In recent work with a community-dwelling older adult sample, poorer episodic memory performance was associated with greater numbers of task-step omission errors and inaccuracies completing complex tasks of daily living, such as filling a medication dispenser and cooking (Schmitter-Edgecombe et al., 2012; Schmitter-Edgecombe and Parsey, 2014).

Mobility refers to the ability to initiate and control movements in space, including such actions as turning, walking and transitions between sitting and standing. A decline in mobility with aging is associated with a decline in overall quality of life (Netuveli et al., 2006). Mobility is sometimes assessed simply by measuring usual walking speed, but a more sensitive measure is Timed Up and Go (TUG), a timed task in which subjects stand up from a chair, walk 3 m, turn 180°, and walk back to sit in the chair again (Podsiadlo and Richardson, 1991). TUG performance is sensitive to mobility deterioration associated with aging (Steffen et al., 2002; Schoene et al., 2014). TUG performance and gait speed have also been shown to relate to visuomotor-dependent measures of executive function, visuospatial working memory and speeded processing (McGough et al., 2011; Donoghue et al., 2012; Martin et al., 2013; Kawagoe et al., 2015). Thus, mobility appears to be strongly associated with visuomotor performance and related functions.

Posture refers to the way the muscles and skeleton are coordinated to maintain an upright orientation against gravity. Older adults have a tendency to carry their heads and necks forward relative to their torsos (Nemmers et al., 2009). This may be caused in part by thoracic hyperkyphosis, a rounding of the upper back that is common in older adults (Griegel-Morris et al., 1992; Quek et al., 2013). We will refer to forward head posture and thoracic hyperkyphosis jointly as “stooped posture” and to their absence as “upright posture.” Stooped posture in older adults has been associated with negative outcomes including falls and increased risk of mortality (Kado et al., 2007). Although stooped posture tends to increase with age, it is not always directly attributable to biomechanical causes such as osteoporosis or arthritis (Katzman et al., 2011). Little research has been done on the cognitive correlates of stooped posture. However, a recent study found that practice of Qigong, a form of mindful movement that includes a focus on upright posture, led to improvement on a test of memory (Tao et al., 2016). Therefore, an association between upright posture and memory is plausible.

We predicted that in a group of generally healthy older adults, mobility would be strongly associated with measures of visuomotor performance and only weakly associated with verbal episodic memory, consistent with previous findings. We also investigated possible associations of stooped posture with both visuomotor performance and verbal episodic memory.

## Materials and Methods

### Subjects

Participants were 162 neurologically healthy adults (114 women and 48 men), aged 50–89 (median 68), with 11–20 years of education (median 16). All were part of a larger study (*N* = 347) investigating the relationship between cognition and everyday functioning across the continuum from healthy aging to dementia. All participants were living independently in the community and were recruited through community health and wellness fairs, physician and local agency referrals, fliers and talks, and prior studies. Initial phone screening with the Telephone Interview for Cognitive Status (TICS; Brandt and Folstein, 2003) was conducted to exclude participants who would have difficulty completing the study protocol (i.e., TICS < 21). Participants who met initial screening criteria completed standardized neuropsychological tests; the current study sample represents those who were classified as healthy adults. Participants were excluded if they had a neurological disorder, met the National Institute on Aging-Alzheimer’s Association workgroups’ core clinical diagnostic criteria for mild cognitive impairment (Albert et al., 2011), or had ever had brain surgery or a stroke. The study was approved by Washington State University’s Institutional Review Board, and subjects gave informed consent before beginning. Data were collected on the Washington State University campus.

### Cognitive Tasks

To assess visuomotor performance, participants were administered the spatial span subtest from the Wechsler Memory Scale-III (Wechsler, 1997) and the design fluency subtest from the Delis-Kaplan Executive Functioning System (Delis et al., 2001). The spatial span subtest uses a three-dimensional board with 10-blocks that the examiner taps at a rate of one block per second producing unique, predetermined patterns of increasing complexity. Participants are administered two trials at each level of complexity and administration is discontinued after the participant misses both trials of a level. The total correct score for spatial span forward, which required participants to immediately duplicate the same patterns from memory, and the total correct score for spatial span backward, which required participants to duplicate the patterns in reverse, were used as dependent variables. The design fluency subtest required participants to quickly draw novel designs by connecting four straight lines in repeated similar arrays of dots (5 or 10 dots) under three conditions: connecting filled dots only, connecting empty dots only and switching between filled and empty dots. Participants were given 60 s per condition. Total correct designs in each condition were summed for a total score.

To test verbal episodic memory, we used the Memory Assessment Scale (Williams, 1991) list learning and memory subtests. Participants were required to learn a list of 12 words. Learning trials were administered until the participant successfully retrieved all 12 words, or six learning trials had been administered. Memory for the word list was later assessed at both a short and long delay. Dependent variables included the serial clustering score from the list learning task and number of the 12 words recalled at short-delay recall and at long-delay recall.

### Predictor Variables

To assess mobility and posture, we measured TUG performance and neck angle. TUG time was assessed with a stopwatch according to established guidelines (Podsiadlo and Richardson, 1991). Scores were inverted to improve homogeneity. Subjects performed a practice trial of the TUG before the data were collected. Neck angle was defined as the angle between a line connecting the tragus to the C7 spinous process and a horizontal line forward. We asked participants to stand as they normally would and look straight ahead while an experimenter took the measurement with an inclinometer attached to a ruler. Two experimenters measured each participant (without observing or consulting each other) to allow assessment of inter-rater reliability. Smaller angles indicate a more flexed neck, indicative of a less upright posture.

We also included a number of physical and demographic measures in our model. Age, sex and years of education were assessed by self-report. Grip strength was measured separately for each hand using a Smedley spring-type hand dynamometer (Lafayette Instrument Co.) and then averaged across hands. Height and weight were measured, and body mass index (BMI) was computed as weight (in kg)/height squared (in m).

### Analysis

Data were analyzed in R (R Development Core Team, 2014). To confirm the grouping of the cognitive tests, we used principal components analysis (PCA) based on a correlation matrix from the raw data, with two components and oblique rotation. We then transformed the predictor variables to *z*-scores and used stepwise multiple regression to determine the combination of predictor variables that produced the best model for each of the two cognitive components. The predictor with the highest correlation was added first, and then other predictors were added one at a time only if they improved the fit of the model. Model fit was evaluated with the Akaike information criterion (AIC), a measure of statistical model quality that takes into account the number of predictor variables (Akaike, 1974). All *p*-values are based on two-tailed tests.

## Results

The inter-rater reliability for the neck angle measure was high (*r* = 0.95), so values were averaged. Tables 1, 2 show the means and standard deviations of the predictor variables and cognitive scores, for men and women. There were significant sex differences for grip strength and neck angle, and for MAS short and long delay scores. Table 3 shows the outcome of the PCA. To test the appropriateness of a two-factor model, we applied the Kaiser rule: only the first two eigenvalues were greater than 1.0, indicating that the first two components each accounted for more information than an average single item (Kaiser, 1960). PCA indicated that the three visuomotor tasks load strongly on the first component (accounting for 30% of the total variance) and the three verbal memory tasks load strongly on the second component (accounting for an additional 26% of total variance).

**Table 1:** **Subject characteristics: mean (SD)**.

Predictor variable	Overall (*N* = 162)	Men (*N* = 48)	Women (*N* = 114)
Age (years)	67.6 (8.9)	68.4 (9.79)	67.3 (8.5)
Years of education	16.6 (2.5)	17.2 (2.7)	16.4 (2.4)
Body mass index	29.0 (8.0)	29.1 (7.9)	29.0 (8.1)
Grip strength (kg)*	31.6 (25.1)	46.4 (19.7)	24.3 (11.3)
TUG time (s)	9.3 (1.9)	9.4 (1.5)	9.2 (2.0)
Neck angle (degrees)*	39.9 (8.0)	36.3 (7.3)	41.3 (7.9)

*Indicates statistically significant difference between men and women (p < 0.05, uncorrected).

**Table 2:** **Cognitive scores: mean (SD)**.

Cognitive task	Overall (*N* = 162)	Men (*N* = 48)	Women (*N* = 114)
Design fluency (items correct)	27.7 (6.5)	26.6 (6.5)	28.1 (6.5)
Forward spatial span	8.3 (1.8)	8.3 (1.7)	8.3 (1.8)
Backward spatial span	7.5 (1.8)	7.7 (1.8)	7.5 (1.8)
MAS: serial clustering (%)	14 (18)	16 (21)	13 (17)
MAS: short delay (# correct)*	11.4 (0.9)	11.1 (1.2)	11.6 (0.7)
MAS: long delay (# correct)*	11.1 (1.2)	10.8 (1.3)	11.2 (1.1)

*Indicates statistically significant difference between men and women (p < 0.05, uncorrected).

**Table 3:** **Principal components analysis (PCA): factor loadings of six cognitive task scores on two components, rotated and extracted with oblique rotation**.

Cognitive task	Visuomotor performance	Verbal episodic memory
Design fluency	**0.698**	0.044
Forward spatial span	**0.801**	0.054
Backward spatial span	**0.799**	−0.073
MAS serial clustering	0.152	**−0.611**
MAS long delay recall	0.038	**0.789**
MAS short delay recall	0.048	**0.746**
Sum of squares loadings	1.79	1.56
Variance explained (%)	30	26

*Factor loadings with an absolute value greater than 0.30 are bolded*.

**Figure 1:**
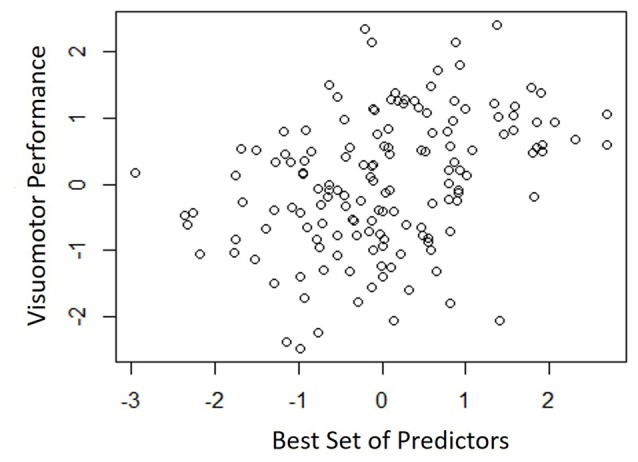
**Best fitting model for visuomotor performance, with age, timed-up-and-go (TUG) speed, and body mass index (BMI) as predictors**.

**Figure 2:**
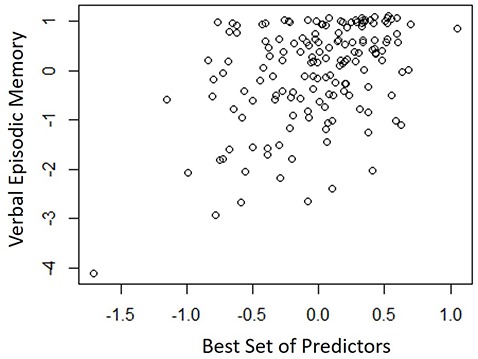
**Best-fitting model for verbal episodic memory, with age, education, sex and neck angle as predictors**.

Table 4 shows the single-variable relations of each of the two cognitive components with age, education, sex, BMI, grip strength, TUG speed and neck angle. Visuomotor performance was significantly correlated with age (*r* = −0.32) and TUG speed (*r* = 0.27), while verbal episodic memory was significantly related to neck angle (*r* = 0.29), age (*r* = 0.22), education level (*r* = 0.17) and sex (*t* = 2.8). Tables 5, 6 show the final results of the multiple regressions. Visuomotor performance (shown in Figure 1) was best explained by a linear combination of age, TUG speed and BMI; *R*^2^ = 0.1585; adjusted *R*^2^ = 0.1411. Episodic memory (shown in Figure 2) was best explained by a combination of age, education, sex and neck angle; *R*^2^ = 0.1848; adjusted *R*^2^ = 0.1633.

**Table 4:** **Single-predictor correlations**.

	Visuomotor performance		Verbal episodic memory	
Predictor	Test statistic	*p*	Test statistic	*p*
Age	**−0.32**	**0.00004**	**−0.22**	**0.007**
Education	0.003	0.97	**0.17**	**0.04**
Sex	0.2	0.84	**2.8**	**0.007**
BMI	0.11	0.19	0.04	0.63
Grip strength	0.12	0.14	−0.11	0.18
TUG speed	**0.27**	**0.0005**	0.10	0.21
Neck angle	0.11	0.19	**0.29**	**0.0002**

*The test statistic was two-tailed Pearson’s correlation coefficient for all variables except sex, where a two-tailed, independent-samples t-test was used. Significant relationships are indicated in bold (p < 0.05, uncorrected)*.

**Table 5:** **Multiple regression for visuomotor performance (*N* = 152)**.

	Beta	Standard error	partial *r*^2^
Age	−0.197	0.085	0.032
TUG speed	0.272	0.086	0.058
BMI	0.016	0.010	0.023

*Predictors were normalized, so beta values indicate the relative weight of each factor in the regression. Total R^2^ = 0.2314, adjusted R^2^ = 0.1975, Akaike information criterion (AIC) = 408.5*.

**Table 6:** **Multiple regression for verbal episodic memory (*N* = 160)**.

	Beta	Standard error	partial *r*^2^
Age	−0.201	0.080	0.034
Education	0.232	0.076	0.050
Sex	0.514	0.174	0.045
Neck angle	0.156	0.083	0.019

*Beta values indicate the relative weight of each factor in the regression. Total R^2^ = 0.1848, adjusted R^2^ = 0.1633, AIC = 426.0*.

## Discussion

This study demonstrated a double dissociation between motor functions and cognitive functions. Poor visuomotor performance was best predicted by a combination of advanced age, high BMI and slow TUG performance, whereas poor verbal episodic memory was best predicted by a combination of advanced age, male sex, low education and forward neck angle. These results confirm previous findings (especially with respect to visuomotor processing and mobility) and add novel information (especially with regard to verbal memory and posture). A novel aspect of the study was our examination of posture on its own, rather than in combination with balance or gait.

### Age and Sex Differences

In this study, men and women were not statistically different in mobility or visuomotor performance, but there were significant sex differences in neck angle and verbal episodic memory. These results are consistent with previous work showing that spatial perception (unlike mental rotation) is similar between men and women (Kaufman, 2007), as is performance on the TUG task (Bohannon, 2006). Our results are also consistent with work showing that verbal episodic memory (unlike some other aspects of memory) is stronger in women than in men (Lewin et al., 2001), and that older men have a greater tendency toward stooped posture than older women (Kado et al., 2007; Mostamand et al., 2013).

### Visuomotor Performance and Mobility

The association shown here between TUG speed and visuomotor performance in healthy older adults is consistent with the literature, which indicates considerable overlap among visuomotor performance, processing speed and executive function (Anguera et al., 2009). For instance, Martin et al. (2013) found that after adjusting for age, sex, medication use, mood, physical activity, and years of education, gait speed was associated with executive function and speeded processing but not with memory. McGough et al. (2011) found that usual (self-selected) gait speed was associated with visually-dependent measures of executive function, even after controlling for age, sex, depression, comorbidities and BMI. This study reported standardized beta coefficients of 0.228 for the Stroop task and 0.256 for Trails B in their regression model. Kawagoe et al. (2015) found that after controlling for age and general cognitive decline, visual-spatial working memory was strongly and significantly correlated with TUG performance (partial *r* of 0.51), but not with manual dexterity. Herman et al. (2011) found that digit span and verbal fluency (both measures of executive function) correlated with TUG score but not with either the Berg Balance Test or the Dynamic Gait Index. The percentage of cognitive variance accounted for by TUG score in the study by Herman et al. (2011) was around 4%, with no correction for covariates. Along similar lines, Donoghue et al. (2012) found in a study of 5000 older adults that slow TUG performance was independently associated with poor executive function and processing speed. After controlling for eight non-cognitive variables, the total contribution of all cognitive variables to TUG speed was about 2%, with the highest standardized regression beta values for Trails A (0.184), Trails B (0.146), and choice reaction time (0.113). How do effect sizes in the present study compare to previous effects relating TUG to cognitive factors? The partial *r-square* value of 0.075 and standardized beta of 0.272 in our regression model are higher than effect sizes seen in prior studies relating TUG to executive function, but not as high as the effect size in the study relating TUG to visual working memory. Thus, our results are in line with what other researchers have found.

### Verbal Episodic Memory and Upright Posture

To our knowledge, this is the first evidence that habitual forward head posture is associated with poor performance on episodic memory tasks, in people of any age. Researchers investigating the relations between posture and cognitive function in older adults have focused almost exclusively on postural control/balance, (e.g., Woollacott and Shumway-Cook, 2002; Horak, 2006; Huxhold et al., 2006). The relatively few studies investigating psychological associations with upright posture have mainly focused on workplace factors such as over-commitment (Bruno Garza et al., 2013) and mental concentration (Shahidi et al., 2013), or emotions such as pride (Stepper and Strack, 1993) and disappointment (Oosterwijk et al., 2009). There is also an interesting body of work investigating how using upright rather than stooped posture might prime cognitive-behavioral modes such as risk-tolerance (Carney et al., 2010), memory for pleasant experiences (Riskind, 1983), memory for words with valence (Michalak et al., 2014), persistence at solving puzzles (Riskind and Gotay, 1982), pain tolerance (Bohns and Wiltermuth, 2012) and willingness to violate social norms (Yap et al., 2013). The possible connection between the effects of posture at a given moment and the influence of habitual posture bears further investigation.

### Possible Causal Relationships

It is not possible to determine a causal relationship based solely on correlational results. However, generally speaking (and pending replicability), there are two possibilities. The first possibility is that some variable affects both verbal memory and posture, while another variable affects visuomotor performance and gait/mobility. For instance, the neurotransmitter acetylcholine is thought to be important both for visuomotor processing (Sarter and Bruno, 1997; Naber et al., 2015) and for gait (Yarnall et al., 2011; Bohnen and Jahn, 2013; Sarter et al., 2015), while dopamine has been shown to be important for posture (Benninger et al., 2015) and episodic memory (Shohamy and Adcock, 2010), especially in older adults (Papenberg et al., 2014; Abdulrahman et al., 2015). Interestingly, dopamine is thought to be less available in men than in women, likely because of the influence of estrogen (Becker, 1999; Mozley et al., 2001; Staley et al., 2001; Jacobs and D’Esposito, 2011). This difference in dopamine levels may account for the higher prevalence of Parkinson’s disease (Gillies et al., 2014) and some types of addictions (Fattore et al., 2014) in men than in women. The dopamine explanation is therefore consistent with the sex differences we observed in both posture and memory. However, dopaminergic therapy in PD does not improve posture as much as it improves bradykinesia and limb rigidity (Bejjani et al., 2000). Furthermore, a decrease in central cholinergic neurons has been linked to memory decline with aging (Davis and Yamamura, 1978; Bartus et al., 1982). Therefore, this causal explanation has serious flaws.

A second possibility is that there is a causal relationship between the correlated factors. It is easy to see how visuomotor ability could affect mobility, as walking to a destination is in part a visuomotor task. A less obvious but nonetheless intriguing idea is that habitual posture might influence memory. The aforementioned body of research showing that short-term posture has immediate effects on memory (Riskind and Gotay, 1982; Michalak et al., 2014) lends credibility to this possibility. Furthermore, a recent clinical study demonstrated that extensive practice of Tai Chi, an activity that includes attention to upright posture, improved memory performance more than walking (Mortimer et al., 2012). Another study found that Baduanjin Qigong, a less strenuous movement form that also involves attention to posture, improved memory as much as Tai Chi (Tao et al., 2016). Together, these studies suggest that it is not (only) the cardiovascular aspect of exercise that leads to memory improvement. An intriguing hypothesis is that upright posture facilitates dopamine production, which in turn facilitates memory.

### Study Limitations and Future Directions

None of the participants in this study had been diagnosed with cognitive impairment. However, numerous longitudinal studies of gait and cognition have found that slow walking speed predicts a decline in cognition (Verghese et al., 2007; Kikkert et al., 2016). Using similar logic, future studies could evaluate whether forward neck posture predicts memory decline in subsequent years. One reason for the popularity of the TUG task is that it is quick and easy to administer, requiring only a stopwatch and a tape measure. The measurement of neck angle is even quicker to administer, and it requires only an inclinometer. Therefore, neck angle (as a way to quantify upright posture) shows promise as a possible early indicator of memory problems. Because TUG speed and neck angle were associated with different aspects of cognition, it might make sense to use both of them to get a broader snapshot of a patient’s cognitive-related motor function than either alone can provide.

Because this is the first study investigating the relationship between habitual posture and memory, our ability to relate our results to previous findings is limited. More work will need to be done to attempt to replicate these results and to understand how these findings, which addressed habitual posture, may be related to findings in which posture was manipulated. In addition, to pursue the dopamine hypothesis, catecholamine tests could be used to evaluate overall dopamine levels. Moreover, it could be important to include interventions that alter posture without affecting the cardiovascular system (e.g., Cohen et al., 2015).

## Conclusion

This study demonstrated a double dissociation between motor functions and cognitive functions. Deficits in visuomotor performance were associated with slow TUG performance, whereas verbal episodic memory deficits were associated with less upright posture. This study provides the first empirical support for a link between postural alignment and memory function in healthy older adults. These results are consistent with evidence that dopamine is important for memory and posture, while acetylcholine is more strongly associated with visuomotor abilities and mobility. Future studies will need to go beyond correlation to search for causal links between cognition and posture.

## Author Contributions

RGC designed the study, performed the analyses, interpreted the data and drafted the manuscript. ANV designed the study and critically revised the manuscript for important intellectual content. MMW provided essential statistical oversight, consultation, and feedback, and critically revised the manuscript for important intellectual content. MS-E designed the study, supervised subject recruitment and data acquisition, and critically revised the manuscript for important intellectual content. All authors approved the final version of the manuscript to be published and agree to be accountable for all aspects of the work in ensuring that questions related to the accuracy or integrity of any part of the work are appropriately investigated and resolved.

## Funding

This work was supported by the National institute of General Medical Sciences at the National Institutes of Health (grant number 5 U54 GM104944: pilot grant to RGC) and by the National Institute of Biomedical Imaging and Bioengineering (grant number R01 EB009675 to MS-E). The contents are solely the responsibility of the authors and do not necessarily represent the official views of NIH.

## Conflict of Interest Statement

The authors declare that the research was conducted in the absence of any commercial or financial relationships that could be construed as a potential conflict of interest.
